# Understanding the effect of Mn^2+^ on Yb^3+^/Er^3+^ co-doped NaYF_4_ upconversion and obtaining the optimal combination of these tridoping

**DOI:** 10.1038/s41598-023-44947-1

**Published:** 2023-10-16

**Authors:** Reza Zarei Moghadam, Hamid Rezagholipour Dizagi, Hans Agren, Mohammad Hossein Ehsani

**Affiliations:** 1 Department of Physics, Faculty of Science, Arak University, Arak, 38156-88349 Iran; 2 Faculty of Physics, Semnan University, P.O. Box: 35195-363, Semnan, Iran; 3 Department of Theoretical Chemistry and Biology, KTH Royal Institute of Technology, 10691 Stockholm, Sweden

**Keywords:** Materials science, Nanoscience and technology, Optics and photonics

## Abstract

In this work, we investigated in detail the upconversion properties of several types of nanoparticles, including NaYF_4_:5%Yb^3+^/30%Mn^2+^, NaYF_4_:40%Mn^2+^/x%Yb^3+^ (x% = 1, 5, 10, 20, 30, and 40), NaYF_4_:2%Er^3+^/x%Mn^2+^ (x% = 20, 30, 40, 50, 60, and 70), NaYF_4_:40%Mn^2+^/x%Er^3+^ (x% = 1, 2, 5, and 10), and NaYF_4_:40%Mn^2+^/1%Yb^3+^/x%Er^3+^ (x% = 0, 2, 5, and 10). We studied their upconversion emission under 980 nm excitation in both pulsed and continuous wave modes at different synthesis temperatures. The nanoparticles were characterized using transmission electron microscopy (TEM), X-ray diffraction (XRD), and photoluminescence (PL) spectroscopy. The doping of Yb^3+^ and Mn^2+^ ions resulted in the nanoparticles assuming cubic and hexagonal crystal structures. The emission intensity increased (106.4 (a.u.*10^3^) to 334.4(a.u.*10^3^)) with increasing synthesis temperature from 120 to 140 °C, while a sharp decrease was observed when the synthesis temperature was increased to 200 °C. The gradual decrease in peak intensity with increasing Mn^2+^ concentration from 20 to 70% was attributed to energy transfer from Mn^2+^ to Yb^3+^. In NaYF_4_:Mn^2+^/Yb^3+^/Er^3+^ UCNPs, increasing the Er^3+^ concentration from 0 to 10% led to the disappearance of the blue, orange, and green emission bands. The intense upconversion luminescence pattern with high spatial resolution indicates excellent potential for applications in displays, biological sensors, photodetectors, and solar energy converters.

## Introduction

Upconversion nanoparticles (UCNPs) possess a range of useful properties, such as low auto-fluorescence from cells, high penetration depth of light, high sensitivity for detection, sharp emission bandwidth, and large anti-Stokes shifts^1–7^, which make them valuable for applications in biological, therapeutic, photonics, and other areas^8–11^. UCNPs typically consist of two types of lanthanide (Ln) dopants, sensitizers and activators, immersed in an inorganic host lattice^12^. Rare-earth (RE) ion-doped UCNPs have attracted increasing interest due to their broad potential applications and unique upconversion (UC) luminescence properties. Specifically, RE-doped UCNPs can convert spectral energy in the infrared, visible, or UV ranges^13–15^.

Sodium yttrium fluoride (NaYF_4_) is one of the most efficient host materials for red, green, and blue UC phosphors^16^. Nanoparticles of NaYF_4_ exist in two structures: hexagonal and cubic, with the hexagonal form typically prepared by a high-energy consumption method at relatively high temperatures and the cubic form prepared by hydrothermal and solvothermal methods at relatively low temperatures^17^. NaYF_4_ has often been applied as a doping host for Ln sensitizers and activators due to its low energy modes (< 400/cm)^18^. Among the Er^3+^(^2^H_11/2_/^4^S_3/2_), Tm_3+_(^3^F_2/3_/^3^H_4_), Pr^3+^(^3^P_1_/^3^P_0_), Eu^3+^(^5^D_1_/^5^D_0_), Dy^3+^(^4^I_15/2_/^4^F_9/2_), and Nd^3+^(^4^F_5/2_/^4^F_3/2_) ions that often employed, Er^3+^-doped NaYF_4_ is recognized as one of the most stable UCNPs upon near-infrared light (NIR) excitations^19–26^. More recently, the incorporation of divalent manganese (Mn^2+^) ions has been recognized to decrease the short-wavelength green emission and increase the long-wavelength red emission due to energy transfer between the RE (often Er^3+^) and Mn^2+^ ions^27^. The Mn^2+^ emission bands, depending on the host surroundings, reside in the region of about 460–700 nm^28^. Previous studies have demonstrated that doping NaYF_4_ with Mn^2+^ ions can lead to strong red fluorescence in UCNPs, making them useful for in *vivo* imaging and drug delivery^29^.

In this paper, we aim to shed further light on this mechanism by investigating the upconversion properties of several types of nanoparticles, including NaYF_4_:5%Yb^3+^/30%Mn^2+^ at different synthesis temperatures, NaYF_4_:40%Mn^2+^/x%Yb^3+^ (x% = 1, 5, 10, 20, 30, and 40), NaYF_4_:2%Er^3+^/x%Mn^2+^ (x% = 20, 30, 40, 50, 60, and 70), NaYF_4_:40%Mn^2+^/x%Er^3+^ (x% = 1, 2, 5, and 10), and NaYF_4_:40%Mn^2+^/1%Yb^3+^/x%Er^3+^ (x% = 0, 2, 5, and 10) nanoparticles under 980 nm excitation in both pulsed and continuous wave modes. We characterized these nanoparticles using transmission electron microscopy (TEM), X-ray diffraction (XRD), and photoluminescence (PL) spectroscopy.

Previous studies have demonstrated that the doping of Mn^2+^ can decrease the non-radiative transition probability, thus improving the intensity of UCNP emission^30–32^. Additionally, among UC materials, α-phase NaYF_4_ is reported as one of the most efficient hosts for enhancing near-infrared (NIR) to single-red band up-conversion when tri-doped with Yb^3+^/Er^3+^/Mn^2+^ ions^33^. It has also been demonstrated that other nanosystems, including NaLuF4:Yb3+/Er3+/Mn2+, MnF2:Yb3+/Er3+, and KMnF3:Yb3+/Er3+, are effective emitters^34^.

In our study, we investigated the upconversion properties of several types of UCNPs to better understand the energy transfer mechanisms among Yb^3+^/Mn^2+^, Mn^2+^/Er^3+^, and Mn^2+^/Er^3+^/Yb^3+^ ions. We found that NaYF_4_ is an efficient host material for red, green, and blue UC phosphors with cubic and hexagonal structures. Er^3+^-doped NaYF_4_ is recognized as one of the most stable UCNPs upon NIR excitations, while Mn^2+^-doped NaYF_4_ can lead to strong red fluorescence in UCNPs. Our results suggest that the doping of Mn^2+^ can decrease the non-radiative transition probability, thus improving the intensity of UCNP emission.

Overall, our findings help to shed further light on the energy transfer mechanisms in UCNPs and highlight the potential applications of these materials in various fields, such as biological sensing, photonics, and solar energy conversion.

## Experiment

### Materials

Yttrium (III) chloride hexahydrate (YCl_3_∙6H_2_O, 99.99%), Erbium (III) chloride hexahydrate (ErCl3∙6H2O, 99.99%), Ytterbium (III) chloride hexahydrate (YbCl_3_∙6H_2_O, 99.99%), Manganese (II) chloride hexahydrate (MnCl_2_∙6H_2_O, 99.99%), Sodium hydroxide (NaOH), oleic acid (OA), ammonium fluoride (NH_4_F), ethanol (pure: 99.9), and cyclohexane were purchased from Sigma-Aldrich company. All chemicals were used as received without further purification.

### Nanoparticle synthesis

#### Synthesis of NaYF_4_:5%Yb^3+^/30%Mn^2+^ nanoparticles

Yb^3+^ and Mn^2+^ co-doped NaYF_4_ nanoparticles were synthesized using a well-established hydrothermal method. NaOH (0.2 M, 1.2 ml) was added to a 50 ml centrifuge tube and stirred for 10 min. A mixture of ethanol (5 ml) and OA (5 ml) was added to NaOH and magnetically stirred for 15 min at room temperature until the solution became uniform and clear. A Ln solution was added to the centrifuge tube (Ln in total is 0.4 mmol): YCl_3_ (0.26 mmol), YbCl_3_ (0.02 mmol) and MnCl_3_ (0.12 mmol). The solution was kept under magnetic stirring for 15 min. Subsequently, 59.26 mg of NH_4_F dissolved in 1.6 ml of deionized water was added to the solution. The final solution was stirred magnetically for 1 h at a temperature of 40 °C. Thereafter, the solution was transferred into a Teflon (PTFE) reactor and sealed with Argon (Ar) gas. The PTFE reactor was inserted into an autoclave and heated at 120 °C, 140 °C, 160 °C, 180 °C, and 200 °C for 8 h. After 8 h, the autoclave was emptied, and the material in the PTFE reactor was cooled to room temperature. The solution was precipitated with ethanol (20 ml), collected by centrifugation (temperature: room temperature, time: 5 min, spin speed: 7500 rpm) washed several times with ethanol (10 ml), and dispersed in cyclohexane (10 ml) for further characterization. Further details of the synthesis of nanoparticles are given in the supplementary file.

### Characterization

The size, shape, structure, and morphological characterization of the as-prepared UCNPs were characterized using a transmission electron microscope (TEM, JEOL, JEM-1400). The optical absorption spectrum in the wavelength range of 300–1000 nm was measured using an Edinburgh FS5 spectrophotometer. UC luminescence (UCL) spectra and decay curves were obtained using an Edinburgh FS5 spectrophotometer equipped with a 980 nm diode laser, operating in pulsed and continuous-wave (CW) mode. The pulse duration for the impact mode was 3 min, the power density of the 980 nm laser was 1 W/cm^2^, and the spot width was 3 mm. 1000 µl of the solution was poured into the cuvette and the cuvette was placed inside the measuring device.

## Results and discussion

### Structure and morphology characterization

**Figure 1:**
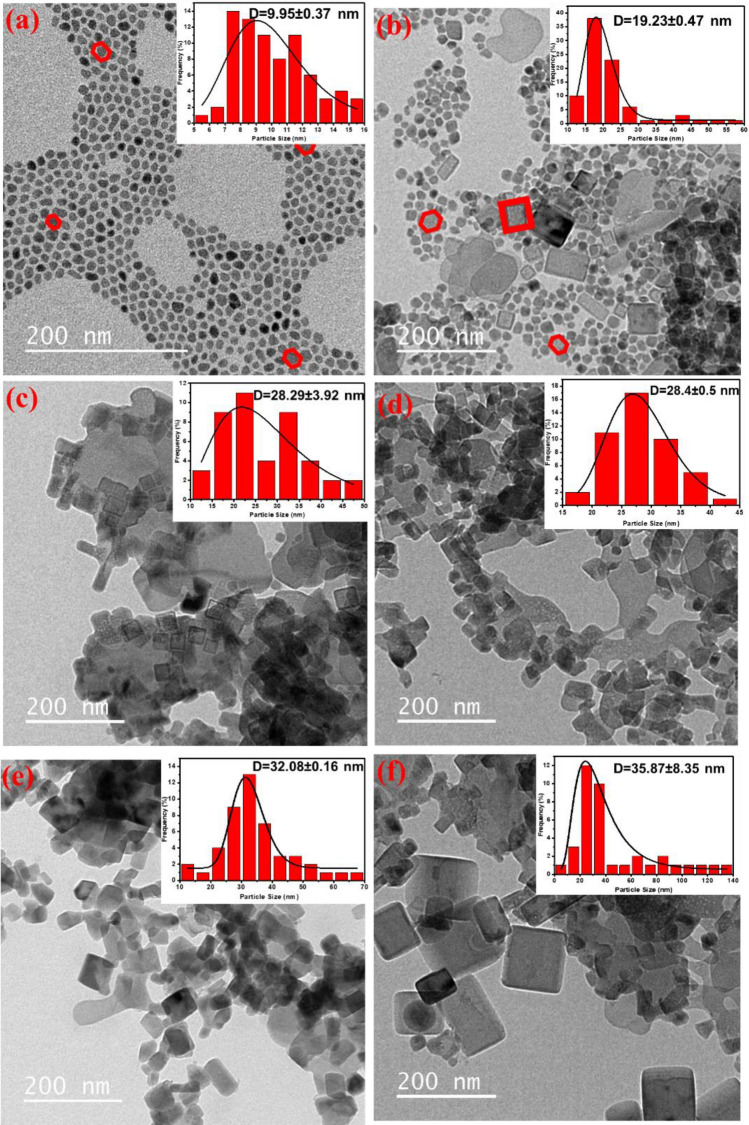
TEM images of the as-synthesized (**a**) NaYF4, (**b**) 30%Mn 5%Yb 120 °C, (**c**) 30%Mn 5%Yb 140 °C, (**d**) 30%Mn 5%Yb 160 °C, (**e**) 30%Mn 5%Yb 180 °C, and (**f**) 30%Mn 5%Yb 200 °C. The analyzed samples were in solution form.

TEM was used to characterize the morphologies of the as-prepared UCNPs. Figure 1 displays TEM images of non-doped NaYF_4_ UCNPs and NaYF_4_: 30% Mn^2+^, 5%Yb^3+^ UCNPs at various synthesis temperatures (120 °C, 140 °C, 160 °C, 180 °C, and 200 °C). The non-doped NaYF_4_ UCNP crystal structure appeared completely hexagonal, while with the addition of Yb^3+^ and Mn^2+^ ions as dopants, the nanoparticles exhibited both cubic and hexagonal crystal structures. The average width of the nanoparticles was determined using the Digimizer software (version 4. 1. 1. 0, MedCalc software) (Fig. 1), and only a minor dispersion of the nanoparticles’ diameters was observed. To estimate the mean diameter of the nanoparticles, the obtained data were fitted with the Log-Normal distribution equation^35^.

1 $$ f\left( D \right) = \left( {\frac{1}{{\sqrt {2\pi } \sigma D}}} \right){\text{exp}}\left[ { - \frac{{ln^{2} \left( {\frac{D}{{D_{0} }}} \right)}}{{2\sigma^{2} }}} \right] $$

2 $$ \left\langle D \right\rangle = D_{0} {\text{exp}}\left( {\frac{{\sigma^{2} }}{2}} \right) $$

3 $$ \sigma_{D} = < D > \left[ {\exp \left( {\sigma^{2} } \right) - 1} \right]^{1/2} $$

Equations (1–3) were used to fit the data and obtain the fitting parameters D_0_ and σ. Additionally, the standard deviation (σ_D_) and mean diameter of the nanoparticles <D> were calculated using the results obtained from the data fitting. As shown in Fig. 1, the diameter of the nanoparticles increased from 19 to 36 nm as the temperature increased from 120 to 200 °C.

**Figure 2:**
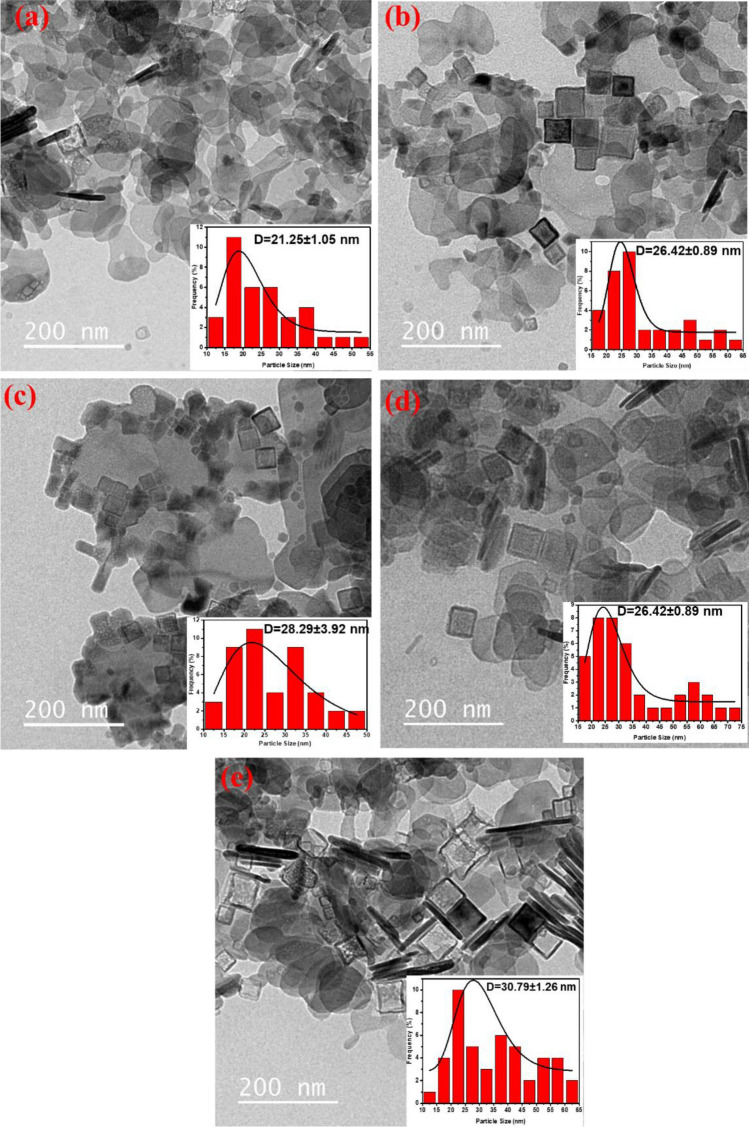
TEM images of NaYF4: Mn^2+^, Yb^3+^ nanocrystals doped with (**a**) 30%, (**b**) 40%, (**c**) 50%, (**d**) 60% and (**d**) 70% Mn^2+^ ions. The analyzed samples were in solution form.

The TEM micrographs in Fig. 2 reveal that the NaYF_4_:5%Yb^3+^/x%Mn^2+^ (x% = 30, 40, 50, 60, 70) nanoparticles are polyhedral in shape with a uniform size. The average diameter of the NaYF4:5%Yb^3+^/x%Mn^2+^ (x% = 30, 40, 50, 60, and 70) nanoparticles was found to be 28 nm. These results suggest that the crystal size of the nanoparticles increases and undergoes continuous and regular changes with the increase in Mn^2+^ content. As Mn^2+^ concentration increases from 30 to 70%, the number and size of the nanoparticles increases, as calculated using Eq. (2). The data demonstrate that the size of the nanoparticles increases from 21 to 30 nm as the Mn^2+^ concentration increases from 30 to 70%. Figure 2 also shows the appearance of several hexagonal hollow nanoparticles as Mn^2+^ concentration increases up to 70%. These observations are consistent with earlier reports^36,37^.

**Figure 3:**
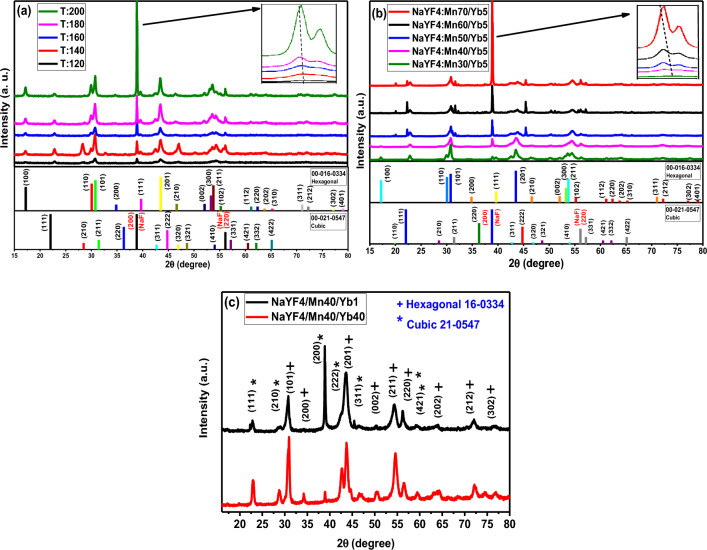
XRD patterns of (**a**) NaYF_4_: 5%Yb^3+^/30%Mn^2+^ UCNPs at different synthesis temperatures (120 °C, 140 °C, 160 °C, 180 °C, 200 °C), (**b**) NaYF4: 5%Yb^3+^/x%Mn^2+^ (x% = 30, 40, 50, 60, 70%) UCNPs, (**c**) NaYF4 UCNPs at different Yb^3+^ concentration (NaYF4:40%Mn^2+^ /1%Yb^3+^ and NaYF4:40%Mn^2+^ /40%Yb^3+^). The analyzed samples were in solution form.

The crystal structure and purity of NaYF_4_:5%Yb^3+^/30%Mn^2+^ UCNPs were studied by analyzing the powder X-ray diffraction (XRD) patterns at different synthesis temperatures. Figure 3a displays XRD patterns of NaYF_4_:5%Yb^3+^/30%Mn^2+^ UCNPs at different synthesis temperatures (120 °C, 140 °C, 160 °C, 180 °C, 200 °C). The XRD patterns of samples prepared at different synthesis temperatures (120 °C, 140 °C, 160 °C, 180 °C, 200 °C) indicate a mixed-phase NaYF_4_ crystal, in agreement with standard patterns of cubic (JCPDS: 00-021-0547) and hexagonal (JCPDS: 00-016-0334). The results demonstrate that increasing the synthesis temperature from 120 to 200 °C leads to an increase in the cubic NaYF_4_ phase peaks and crystallinity, which is consistent with the TEM images. Additionally, the zoomed image (Fig. 3a) shows that when the synthesis temperature is increased to 200 °C, the diffraction peak (111) slightly shifts to the lower-angle side, which can be attributed to the substitution of Y^3+^ ions by smaller Mn^2+^ ions in the host lattice^36^. Figure 3b shows the XRD patterns of NaYF_4_:5%Yb^3+^/x%Mn^2+^ (x% = 30, 40, 50, 60, 70) UCNPs. The XRD patterns indicate a mixed-phase NaYF4 crystal and are consistent with the standard patterns of cubic (JCPDS: 00-021-0547) and hexagonal (JCPDS: 00-016-0334) phases. When the concentration of Mn^2+^ is increased from 30 to 40%, the XRD pattern shows a cubic NaYF_4_ phase. However, when the concentration of Mn^2+^ is further increased from 40 to 70%, the hexagonal phase reappears. Also, by increasing the concentration of Mn^2+^ ions from 40 to 70%, the cubic phase peaks increase. Additionally, increasing the concentration of Mn^2+^ ions from 40 to 70% leads to an increase in cubic phase peaks, which is consistent with the TEM images. The zoomed image (Fig. 3b) shows that with an increase in the concentration of Mn^2+^ ions, the diffraction peak (111) slightly shifts to the lower angle side due to the substitution of Y^3+^ ions by smaller Mn^2+^ ions in the host lattice^36^. Figure 3c displays the XRD patterns of NaYF_4_ UCNPs at different Yb^3+^ concentrations (NaYF_4_:40%Mn^2+^/1%Yb^3+^ and NaYF_4_:40%Mn^2+^/40%Yb^3+^). The XRD patterns indicate a mixed-phase NaYF_4_ crystal, consistent with standard patterns of hexagonal (JCPDS: 00-016-0334) and cubic (JCPDS: 00-021-0547) phases. The results show that increasing the Yb^3+^ concentration from 1 to 40% leads to a decrease in crystallinity. Moreover, with an increase in Yb^3+^ concentration, the cubic NaYF_4_ phase increases while the hexagonal phase decreases. Therefore, the NaYF_4_:40%Mn^2+^/1%Yb^3+^ sample was chosen as the main sample.

In summary, XRD is a powerful technique that allows for the analysis of crystal structure and purity. The XRD patterns of NaYF_4_: 5%Yb^3+^/30%Mn^2+^ UCNPs, NaYF_4_: 5%Yb^3+^/x%Mn^2+^ (x% = 30, 40, 50, 60, 70) UCNPs, and NaYF_4_ UCNPs at different Yb^3+^ concentrations were analyzed, and the results indicate mixed-phase NaYF_4_ crystals with standard patterns of cubic and hexagonal phases. The crystallinity and phase composition were found to be dependent on temperature and dopant concentration, which is consistent with the TEM images.

**Figure 4:**
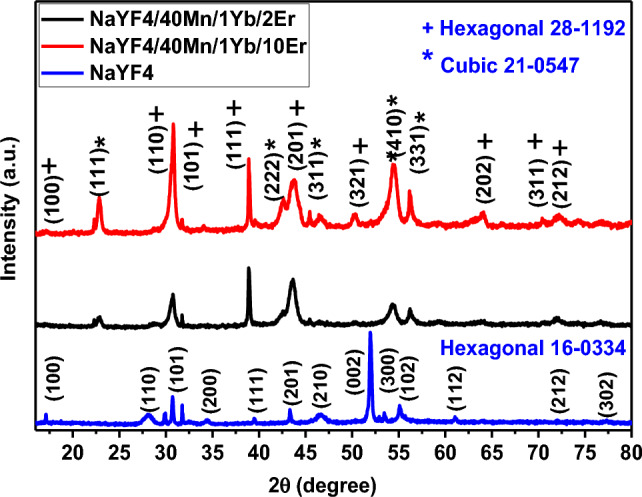
XRD patterns of NaYF_4_, NaYF_4_:1%Yb^3+^/40%Mn^2+^/2%Er^3+^ and NaYF_4_:1%Yb^3+^/40%Mn^2+^/10% Er^3+^ UCNPs. The analyzed samples were in solution form.

Figure 4 displays XRD patterns of NaYF_4_ and NaYF_4_ UCNPs at different Er^3+^ concentrations (NaYF_4_:1%Yb^3+^/40%Mn^2+^/2%Er^3+^ and NaYF_4_:1%Yb^3+^/40%Mn^2+^/10% Er^3+^). The XRD pattern of the UCNPs can be assigned to a mixed-phase NaYF_4_ crystal with the standard pattern of hexagonal (JCPDS: 00-016-0334) and cubic (JCPDS: 00-021-0547). No significant additional diffraction peaks were detected, even when the Er^3+^ feeding content was as high as 10 mol%. The results indicate that the crystallinity decreased with increasing Er^3+^ concentration from 2 to 10%. Additionally, with an increased Er^3+^ concentration, the XRD peak intensity increases in the cubic and hexagonal phases. Consequently, the NaYF_4_:1%Yb^3+^/40%Mn^2+^/10% Er^3+^ UCNP sample was chosen as the primary sample.

### Determination of optimum synthesis temperature for NaYF_4_: 5% Yb^3+^/30% Mn^2+^ nanoparticles

**Figure 5:**
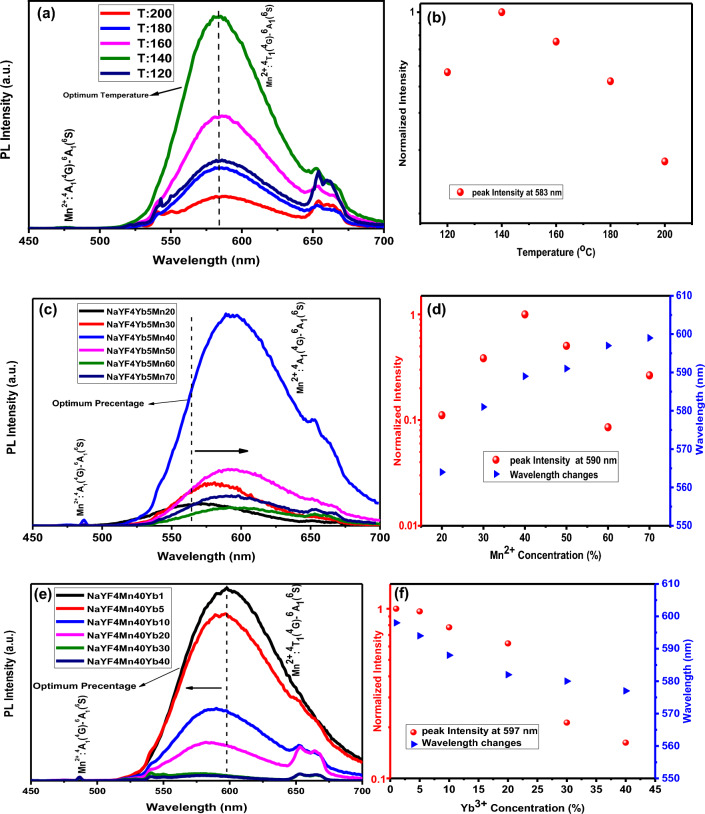
(**a**) UCL emission spectra of NaYF_4_:5%Yb^3+^/30%Mn^2+^ nanoparticles with different synthesis temperatures (120 °C, 140 °C, 160 °C, 180 °C and 200 °C) and (**b**) log intensity versus synthesis temperature under 980 nm CW excitation. (**c**) UCL emission spectra of NaYF_4_:5%Yb^3+^/x%Mn^2+^ (x% = 20, 30, 40, 50, 60 and 70%) nanoparticles and (**d**) log intensity versus Mn^2+^ concentration under 980 nm CW excitation. (**e**) UCL emission spectra of NaYF_4_:40% Mn^2+^/x%Yb^3+^ (x% = 1, 5, 10, 20, 30 and 40%) nanoparticles and (**f**) log intensity versus Yb^3+^ concentration under 980 nm CW excitation. The pulse duration for the impact mode was 3 min, the power density of the 980 nm laser was 1 W/cm^2^, and the spot width was 3 mm. 1000 µl of the solution was poured into the cuvette and the cuvette was placed inside the measuring device.

The UCL spectra of the different synthesis temperatures (120 °C, 140 °C, 160 °C, 180 °C, 200 °C) of NaYF_4_: 5% Yb^3+^/30% Mn^2+^ nanoparticles were measured using CW 980 nm excitation. Figure 5a displays the UCL emission spectra of these nanoparticles, revealing a relatively strong emission band at 583 nm and a weaker emission band at 575 nm under 980 nm excitation, corresponding to Mn^2+^: ^4^A_1_ (^4^G) → ^6^A_1_ (^6^S) and Mn^2+^: ^4^T_1_ (^4^G) → ^6^A_1_ (^6^S) respectively, as shown in Fig. 5a. Our objective was to determine the optimal synthesis temperature for subsequent experiments, and thus, the relationship between reaction temperature and emission intensity was crucial. As depicted in Fig. 5a, the emission intensity increased as the synthesis temperature increased from 120 to 140 °C, but significantly decreased as the synthesis temperature increased to 200 °C. Figure 5b illustrates the log intensity different synthesis temperatures (120 °C, 140 °C, 160 °C, 180 °C, 200 °C) nanoparticles, indicating that the synthesis temperature of 140 °C resulted in the highest emission intensity among all samples, which was considered the optimum synthesis temperature. These observations were attributed to the shrinking host lattice and the decreased non-radiative relaxation processes, consistent with a previous study^38^.

### Optical properties of NaYF_4_: Yb^3+^/Mn^2+^ under 980 nm excitation

Figure 5c and e present the UCL yellow emission spectra of NaYF_4_: Yb^3+^/Mn^2+^ nanoparticles at their optimum synthesis temperature (140 °C). The UCL spectra were measured using CW 980 nm excitation, revealing a visible area to yellow UCL emission. The pulse duration for the impact mode was 3 min, the power density of the 980 nm laser was 1 W/cm^2^, and the spot width was 3 mm. 1000 µl of the solution was poured into the cuvette and the cuvette was placed inside the measuring device. The UCL spectrum consisted of a weak emission peak at 487 nm, corresponding to the d–d transition [Mn^2+^: ^4^A_1_ (^4^G) → ^6^A_1_ (^6^S)] and a broadband emission peak at approximately 590 nm, corresponding to Mn^2+^: ^4^T_1_ (^4^G) → ^6^A_1_ (^6^S). Figure 5c illustrates the concentration-dependent UCL emission of NaYF_4_: 5Yb^3+^/x%Mn^2+^ (x% = 20, 30, 40, 50, 60 and 70), with the optimal doping concentration of Mn^2+^ determined to be x = 40%. Figure 5d displays the log intensity versus Mn^2+^ concentration, indicating an increase in emission intensity as the Mn^2+^ percentage increased from 20 to 40%, followed by a sharp decrease as the Mn^2+^ percentage increased to 70%. The gradual decrease in peak intensity with increasing Mn^2+^ concentration was attributed to energy transfer from Mn^2+^ to Yb^3+^^39^. Additionally, with increasing Mn^2+^ concentration, the peak position shifted from 563 nm (yellow) to 593 nm (orange), confirming this energy transfer. Figure 5e shows the PL intensity versus Yb^3+^ concentration, indicating a decrease in emission intensity as the Yb^3+^ percentage increased from 1 to 40%, with the optimal doping concentration of Yb^3+^ determined to be x = 1%^33^. The decrease in peak intensity with increasing Yb^3+^ concentration was attributed to an energy transfer from Yb^3+^ to Mn^2+^^39^. Furthermore, with increasing Yb^3+^ concentration, the peak position shifted from 599 nm (orange) to 573 nm (yellow), confirming this energy transfer (Fig. 5f). Visible photons from the excited Yb^3+^-Mn^2+^ pairs were released via the sequential ground-state absorption (GSA, |^2^F_7/2_, ^6^A_1_(^6^S) > →|^2^F_5/2_, ^6^A_1_(^6^S)>) and excited-state absorption (ESA, |^2^F_5/2_, ^6^A_1_(^6^S) > →|^2^F_7/2_, ^4^T_1_(^6^S)>) processes.

### Luminescence decay time of NaYF_4_: Yb^3+^/Mn^2+^ nanoparticles under 980 nm excitation

**Figure 6:**
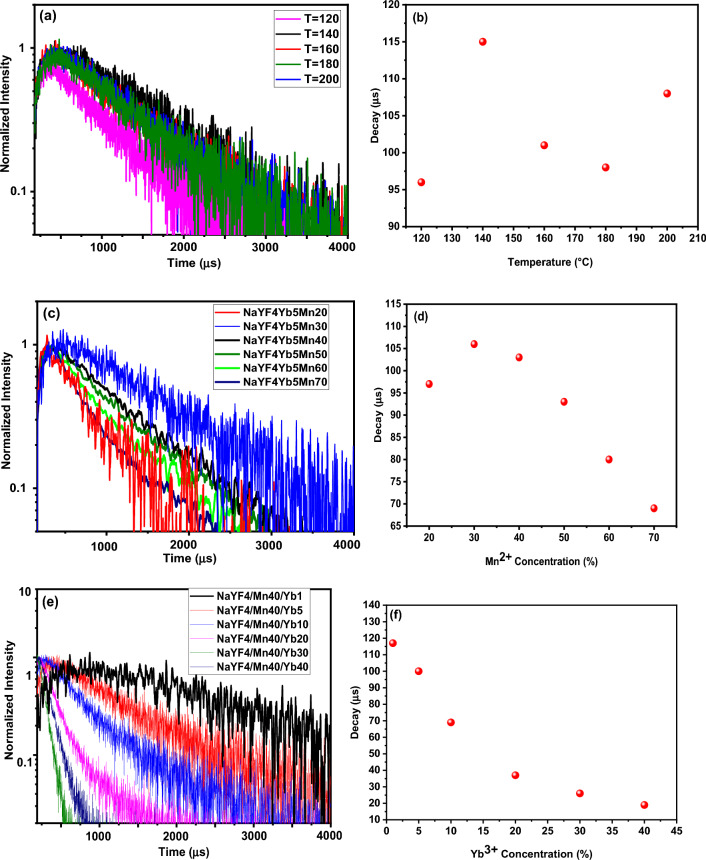
(**a**) Lifetimes spectra of NaYF_4_:5%Yb^3+^/30%Mn^2+^ nanoparticles with different synthesis temperatures (120 °C, 140 °C, 160 °C, 180 °C and 200 °C) and (**b**) amount of decay under 980 nm pulsed excitation. (**c**) Lifetimes spectra of NaYF_4_:5%Yb^3+^/x%Mn^2+^ (x% = 20,30, 40, 50, 60 and 70) nanoparticles, (**d**) amount of decay under 980 nm pulsed excitation. (**e**) Lifetimes spectra of NaYF_4_:40% Mn^2+^/x%Yb^3+^ (x% = 1, 5, 10, 20, 30 and 40) nanoparticles, (**f**) amount of decay under 980 nm pulsed excitation. The power density and spot diameter of the used 980-nm laser are 10 W/cm^2^ and 3.0 mm, respectively. Luminescence decay times were measured in the red wavelength region. The analyzed samples were in solution form.

To provide further evidence for the role that temperature plays in enhancing UC emission, decay curves of the UCNPs are presented in Fig. 6a–f. Decay curves were fitted with the following formula proposed by Nakazawa^40^:

$$ \tau_{m} = \frac{{\int_{0}^{\infty } {tI\left( t \right)dt} }}{{\int_{0}^{\infty } {I\left( t \right)dt} }} $$

where τ_m_ is the effective decay time constant, and I(t) is the intensity at time t.

Figure 6 displays the UC luminescence decay curves of (a) NaYF4:5%Yb^3+^/30%Mn^2+^ nanoparticles at different synthesis temperatures (120 °C, 140 °C, 160 °C, 180 °C and 200 °C), (b) the amount of decay under 980 nm pulsed excitation, (c) NaYF4:5%Yb^3+^/x% Mn^2+^ (x% = 20, 30, 40, 50, 60 and 70) nanoparticles decay, (d) the amount of decay under 980 nm pulsed excitation, (e) NaYF4:40% Mn^2+^/x%Yb^3+^ (x% = 1, 5, 10, 20, 30 and 40) nanoparticles decay, and (f) the amount of decay under 980 nm pulsed excitation. The pulse duration for the impact mode was 3 min, the power density of the 980 nm laser was 10 W/cm^2^, and the spot width was 3 mm. 1000 µl of the solution was poured into the cuvette and the cuvette was placed inside the measuring device.

Figures 6a and b show that the luminescence lifetimes increase from 96 to 115 µs as the synthesis temperature rises from 120 to 140 °C, while as the synthesis temperature increases from 140 to 200 °C, the luminescence lifetimes decrease. Therefore, the NaYF4:5%Yb^3+^/30%Mn^2+^ nanoparticles with a synthesis temperature of 140 °C, showing the longest luminescence lifetime, were selected as the main sample. As previously demonstrated, a synthesis temperature of 140 °C is the optimal synthesis temperature for producing samples. Figure 6c and d reveal that by increasing the Mn^2+^ concentration from 20 to 70%, the luminescence lifetimes of the Yb^3+^: ^2^F_5/2_ level first increase and then decrease significantly. The luminescence lifetimes for Yb^3+^ were found to decrease monotonically with increasing Mn^2+^ concentration, providing evidence for efficient energy transfer from Yb^3+^ to Mn^2+^ ions^37^. These time-decay measurements are consistent with the UCL emission spectra. Figure 6e and f illustrate that by increasing Yb^3+^ concentration from 1 to 40%, the luminescence lifetimes of the Yb^3+^: ^2^F_5/2_ level decrease significantly from 116 to 15 µs. The luminescence lifetimes for NaYF4: 40%Mn^2+^/x%Yb^3+^ (x% = 1, 5, 10, 20, 30 and 40) UCNPs were found to decrease monotonically with increasing Yb^3+^ concentration, providing evidence for efficient energy transfer from Yb^3+^ to Mn^2+^ ions^37^. Our main objective was to find a combination of Yb^3+^ and Mn^2+^ that had the longest luminescence lifetime. Therefore, a sample of NaYF4: 40%Mn^2+^/1% Yb^3+^ was selected as the main sample and used in the subsequent steps. These results are in good agreement with the UCL emission spectra and observed structural properties.

### Optical properties of NaYF_4_:Er^3+^/Mn^2+^ nanoparticles under 980 nm excitation

**Figure 7:**
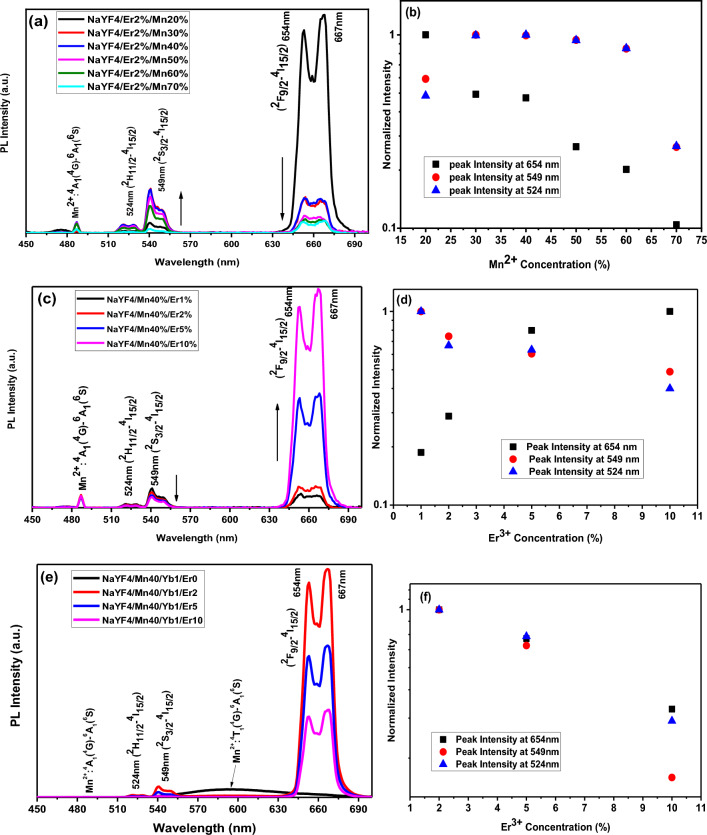
(**a**) UCL emission spectra of NaYF_4_: 40% Mn^2+^/x%Er^3+^ (x% = 1, 2, 5 and 10) nanoparticles and (**b**) log intensity versus Er^3+^ concentration under 980 nm CW excitation. (**c**) UCL emission spectra of NaYF_4_: 2% Er^3+^/x%Mn^2+^ (x% = 20, 30, 40, 50, 60 and 70) nanoparticles (**d**) log intensity versus Mn^2+^ concentration under 980 nm CW excitation. (**e**) UCL emission spectra of NaYF_4_: 1% Yb^3+^/40% Mn^2+^/x%Er^3+^ (x% = 0, 2, 5, and 10) nanoparticles (**f**) log intensity versus Er^3+^ concentration under 980 nm CW excitation. NaYF_4_:Yb^3+^/Mn^2+^ nanoparticles emitted relatively strong emission band at 575 nm. The pulse duration for the impact mode was 3 min, the power density of the 980 nm laser was 1 W/cm^2^, and the spot width was 3 mm. 1000 µl of the solution was poured into the cuvette and the cuvette was placed inside the measuring device.

To investigate the energy transfer between Mn^2+^ and Er^3+^ and the luminescence properties of NaYF4: Er^3+^/Mn^2+^, the UCL emission spectra of synthetic samples were measured under CW 980 nm excitation. The pulse duration for the impact mode was 3 min, the power density of the 980 nm laser was 1 W/cm^2^, and the spot width was 3 mm. 1000 µl of the solution was poured into the cuvette and the cuvette was placed inside the measuring device. Figure 7a displays the UCL emission spectra of NaYF_4_: 5%Er^3+^/x%Mn^2+^ (x% = 20, 30, 40, 50, 60 and 70) nanoparticles. The UCL spectra consist of four emission bands centered at 486 nm (blue), 524 (green), 549 nm (green), and 654 nm (red), corresponding to the transitions of ^4^A_1_ (^4^G) → ^6^A_1_ (^6^S), ^2^H_11/2_ → ^4^I_15/2_, ^2^S_3/2_ → ^4^I_15/2_ and ^2^F_9/2_ → ^4^I_15/2_, respectively. The UCL spectra in Fig. 7a demonstrate that the green and blue emissions increase when the Mn^2+^ concentration increases to 40%, but then decrease when it increases to 70%. Additionally, the red emission decreases when the Mn^2+^ concentration increases from 20 to 70%^41^. These results suggest that increasing the Mn^2+^ concentration leads to a continuous decrease in the emission intensity of Er^3+^, indicating the possibility of energy transfer from Er^3+^ ions to Mn^2+^^42^.

To gain further insight into the mechanism of Mn^2+^ doped NaYF_4_: 2%Er^3+^ nanoparticles, the log intensity versus Mn^2+^ concentration for 524 nm, 549 nm, and 654 nm was calculated and presented in Fig. 7b. The green emission intensity increases as the Mn^2+^ concentration increases from 20 to 40%, but then decreases as it increases to 70%. Moreover, the red emission intensity decreases as the Mn^2+^ concentration increases from 20 to 70%, confirming the role of Mn^2+^ in enhancing the green and blue emission and suppressing the red emission in the NaYF_4_: Er^3+^/Mn^2+^ system^41^.

Figure 7c shows the UCL emission spectra of the NaYF4: 40Mn^2+^/x%Er^3+^ (x% = 1, 2, 5 and 10) nanoparticles. The spectra consist of four emission bands centered at 486 nm (blue), 524 nm (green), 549 nm (green), and 654 nm (red), corresponding to the transitions of ^4^A_1_ (^4^G) → ^6^A_1_ (^6^S), ^2^H_11/2_ → ^4^I_15/2_, ^2^S_3/2_ → ^4^I_15/2_ and ^2^F_9/2_ → ^4^I_15/2_, respectively. The results indicate that as the Er^3+^ concentration increases from 1 to 10%, the green and blue emissions decrease, while the red emission increases^41^. This finding suggests that increasing the Er^3+^ concentration leads to a continuous decrease in the emission intensity of Mn^2+^, indicating the possibility of energy transfer from Mn^2+^ ions to Er^3+^^42^.

To gain a deeper understanding of the mechanism of Er^3+^ doped NaYF_4_: 40%Mn^2+^ nanoparticles, the log intensity versus Er^3+^ concentration for 524 nm, 549 nm, and 654 nm was calculated and presented in Fig. 7d. The results show that as the Er^3+^ concentration increases from 1 to 10%, the green UC emission bands at 524 nm (Er^3+^:^2^H_11/2_–^4^I_15/2_) and 549 nm (Er^3+^:^4^S_3/2_–^4^I_15/2_) and blue UC emission bands at 486 nm (Mn^2+^: ^4^A_1_ (^4^G) → ^6^A_1_) decrease, while the red UC emission bands at 654 nm (Er^3+^: ^2^F_9/2_ → ^4^I_15/2_) increase, confirming the role of Er^3+^ in reducing the green and blue emission and enhancing the red emission in NaYF_4_: Er^3+^/Mn^2+^ systems^41^. First of all, the possible energy transfer from Er^3+^ to Mn^2+^–Yb^3+^ dimer, which contributes to the emission intensity as 524 and 549 nm decreased, can be explained by the following mechanisms: ^4^S_3/2_ (Er^3+^) ^2^F_7/2_, ^6^A_1_ (Mn^2+^–Yb^3+^ dimer) ^4^I_15/2_ (Er^3+^) ^2^F_7/2_, ^4^T_1_ (Mn^2+^–Yb^3+^ dimer). Secondly, the increased luminescent centers lead the emission intensity at 657 nm (Er^3+^: ^4^F_9/2_–^4^I_15/2_) may be energy transfer from Mn^2+^–Yb^3+^ dimer to Er^3+^ ions. The mechanism of energy transfer from Mn^2+^–Yb^3+^ dimer to Er^3+^ ions was proposed as follows: ∣^2^F_7/2_, ^4^T_1_〉 (Mn^2+^–Yb^3+^ dimer) + ^4^I_15/2_ (Er^3+^)–^4^F_9/2_ (Er^3+^) + ∣^2^F_7/2_, ^6^A_1_〉 (Mn^2+^–Yb^3+^ dimer), so the energy transfer bridge was constituted by the energy transfer process between Er^3+^ ion and the Mn^2+^–Yb^3+^ dimer.

To further investigate the impact of Er^3+^ doping on the upconversion luminescence (UCL) properties of Yb^3+^/Er^3+^/Mn^2+^ triply-doped NaYF_4_ nanoparticles, the emission intensity versus wavelength of NaYF_4_: 1%Yb^3+^/40%Mn^2+^/x%Er^3+^ (x% = 0, 2, 5, and 10) nanoparticles at the optimum synthesis temperature is presented in Fig. 7e. When excited at 980 nm, five UCL bands at 486 nm, 524 nm, 549 nm, 595 nm, and 654 nm are detected, which are attributed to the ^4^A_1_ (^4^G) → ^6^A_1_ (^6^S), ^2^H_11/2_ → ^4^I_15/2_, ^2^S_3/2_ → ^4^I_15/2_, ^4^T_1_ (^4^G) → ^6^A_1_ (^6^S) and ^2^F_9/2_ → ^4^I_15/2_ transitions, respectively. The results show that as the Er^3+^ concentration increases from 0 to 10%, all emission bands, blue, orange, and green, disappear for the NaYF_4_:Mn^2+^/Yb^3+^/Er^3+^ nanoparticles. This single-band UCL emission can be attributed to a non-radiative energy transfer from the ^4^S_3/2_ and ^2^H_11/2_ levels of Er^3+^ to the ^4^T_1_ and ^4^A_1_ levels of Mn^2+^^43^. The disappearance of green emissions with increasing Er^3+^ concentration suggests an efficient exchange energy transfer process between Mn^2+^ and Er^3+^ ions, which is attributed to their proximity and nearly perfect overlap of energy levels in the host lattices^33,44–46^. Additionally, it is noteworthy that the red emission intensity gradually decreases with the increase in Er^3+^ concentration due to concentration quenching between neighboring Er^3+^ ions^43^.

In Fig. 7c, the luminescence of sample NaYF_4_: 10%Er^3+^, 40% Mn^2+^ is the highest. But when the three dopants, namely; manganese, yttrium and ytterbium are added to NaYF_4_, sample NaYF_4_: 1% Yb^3+^/40% Mn^2+^/2%Er^3+^ is the highest, so we consider sample NaYF_4_: 1% Yb^3+^/40% Mn^2+^/2%Er^3+^ as the optimal sample (Fig. 7e).

To better comprehend the UCL mechanism, the log intensity versus Er^3+^ concentration for NaYF_4_: 1%Yb^3+^/40%Mn^2+^/x%Er^3+^ (x% = 0, 2, 5, 10) nanoparticles under CW 980 nm excitation is calculated and presented in Fig. 7f. The results show that as the Er^3+^ concentration increases, the green emission disappears and the red emission intensity decreases.

**Figure 8:**
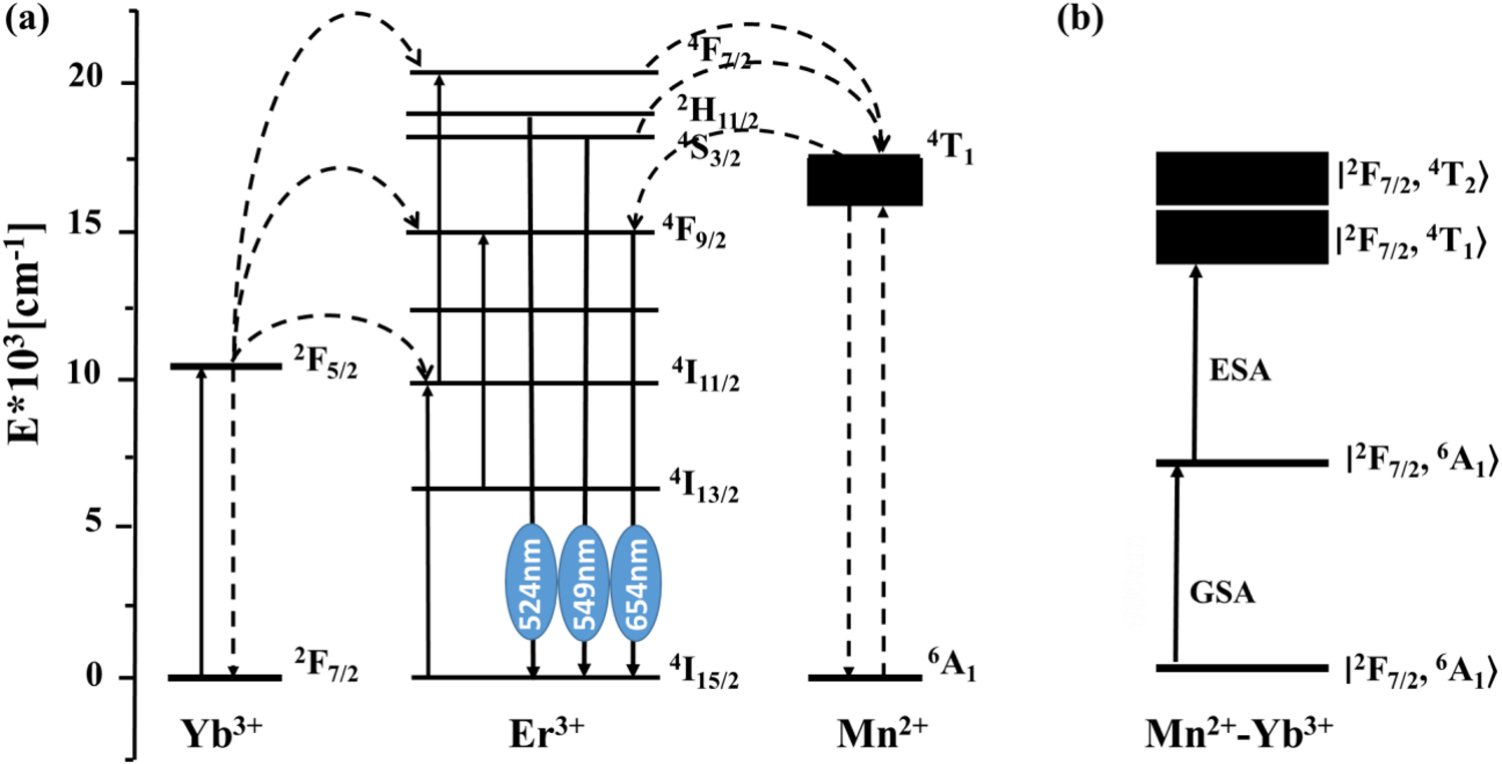
(**a**) Schematic of energy level diagrams of NaYF4:Yb^3+^ /Mn^2+^/Er^3+^ and the proposed mechanism of the UC process under the excitation of 980 nm. (**b**) Schematic of energy level diagrams of Mn^2+^–Yb^3+^ dimer.

To further investigate the effect of Mn^2+^ on the upconversion luminescence (UCL) properties of Yb^3+^/Mn^2+^/Er^3+^ triply-doped NaYF_4_ nanoparticles, a diagram of energy levels and corresponding energy transfer mechanisms is presented in Fig. 8a. When Mn^2+^ ions are introduced into NaYF_4_: Yb^3+^/Er^3+^, a new energy transfer process between Er^3+^ and Mn^2+^ is induced under the excitation of 980 nm CW laser. This process leads to a decrease in the radiative transition rate of Er^3+^:H_11/2_ and Er^3+^: S_3/2_ levels to the ground state, while the population density of Mn^2+^:^4^T_1_ increases due to the resonance energy transfer. Subsequently, a back-energy transfer from ^4^T_1_ of Mn^2+^ to the ^4^F_7/2_ level of Er^3+^ leads to an enhancement in the red emission. Here, the direct multi-phonon relaxation process and the indirect energy transfer process of (F_5/2_(Yb^3+^), ^4^I_13/2_(Er^3+^)) → (^2^F_7/2_(Yb^3+^), ^7^I_9/2_(Er^3+^)) are expected to have a minor contribution to the population of ^4^S_3/2_ energy level in Er^3+^ ions. It is noteworthy that the Er^3+^: F_9/2_ lifetime is shorter than that of Mn^2+^:^4^T_1_, which explains why no orange luminescence is observed corresponding to the Mn^2+^: ^4^T_1_ → ^6^A_1_ transition. A similar mechanism has been discussed in NaYF_4_ by Zhangyu Huang et al.^34^. The Schematic of energy level diagrams of Mn^2+^–Yb^3+^ dimer is shown in Fig. 8b. For the Mn^2+^–Yb^3+^ dimer, the sensitization through the Mn^2+^–Yb^3+^ dimer complex entails both ground state absorption (GSA) and excited state absorption (ESA). The Mn^2+^–Yb^3+^ dimer ground state is represented by ∣^2^F_7/2_, ^6^A_1_〉, the intermediate excited state in the NIR by ∣^2^F_5/2_, ^6^A_1_〉, and the relevant higher excited states by ∣^2^F_7/2_, ^4^T_1_〉 and ∣^2^F_7/2_, ^4^T_2_〉.

### Luminescence decay time of NaYF_4_: 1% Yb^3+^/40% Mn^2+^/x%Er^3+^ (x% = 0, 2, 5, and 10) nanoparticles under 980 nm excitation

**Figure 9:**
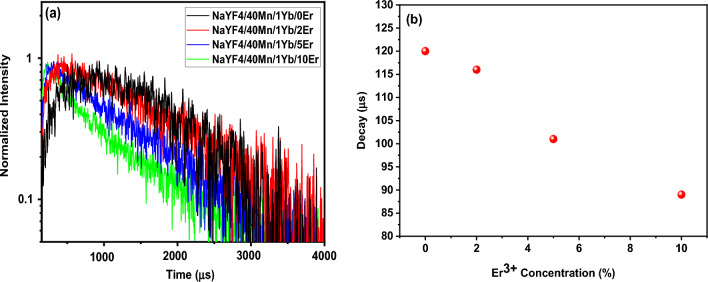
(**a**) Lifetimes spectra of NaYF_4_: 1% Yb^3+^/40% Mn^2+^/x%Er^3+^ (x% = 0, 2, 5, and 10) nanoparticles, (**b**) amount of decay under 980 nm pulsed excitation. The power density and spot diameter of the used 980-nm laser are 10 W/cm^2^ and 3.0 mm, respectively. Luminescence decay times were measured in the red wavelength region. The analyzed samples were in solution form.

To provide further evidence of the role played by Er^+3^ in enhancing UC emission, Fig. 9a and b presents the decay curves of the UCNPs. The pulse duration for the impact mode was 3 min, the power density of the 980 nm laser was 10 W/cm^2^, and the spot width was 3 mm. 1000 µl of the solution was poured into the cuvette and the cuvette was placed inside the measuring device. Figure 9a shows the lifetime spectra of NaYF_4_: 1% Yb^3+^/40% Mn^2+^/x% Er^3+^ (x% = 0, 2, 5, and 10) nanoparticles, while Fig. 9b shows the amount of decay under 980 nm pulsed excitation. The luminescence lifetimes of NaYF_4_: Yb^3+^/Mn^2+^/Er^3+^ were found to decrease monotonically with increasing Er^3+^ concentration. This provides evidence for efficient energy transfer from Yb^3+^ to Mn^2+^ ions^38^. The decrease in lifetime is likely due to a competitive effect between the energy transfer of Er^3+^ and Mn^2+^ ions and the decrease in radiative transition probability resulting from increased local symmetry after Er^3+^ doping. The former prolongs the lifetime, while the latter decreases it.

## Conclusions

In this study, we investigated the UC energy transfer mechanism of Yb^3+^/Mn^2+^/Er^3+^ tri-doped uniform cubic NaYF_4_ nanoparticles under 980 nm excitation in pulsed or continuous-wave modes. Firstly, we synthesized NaYF_4_:5%Yb^3+^/30%Mn^2+^ nanoparticles at different synthesis temperatures, and the optimal synthesis temperature of 140 °C was selected for subsequent experiments. Next, we studied NaYF_4_:5%Yb^3+^ nanoparticles with varying x%Mn^2+^ (x% = 20, 30, 40, 50, 60, 70) concentration and NaYF_4_:40% Mn^2+^ nanoparticles with varying x%Yb^3+^ (x% = 1, 5, 10, 20, 30, 40) concentration. Finally, we synthesized NaYF_4_:40% Mn^2+^/1%Yb^3+^ nanoparticles with different x%Er^3+^ (x% = 0, 2, 5, 10) concentrations by selecting the optimal Mn^2+^ and Yb^3+^ concentrations. The UCL spectra of NaYF_4_: 1% Yb^3+^/40% Mn^2+^/x%Er^3+^ (x% = 0, 2, 5, and 10) nanoparticles exhibited four emission bands centered at 486 nm (blue), 524 nm (green), 549 nm (green), and 654 nm (red), corresponding to the transitions of ^4^A_1_ (4G) → ^6^A_1_ (^6^S), ^2^H_11/2_ → ^4^I_15/2_, ^2^S_3/2_ → ^4^I_15/2_, and ^2^F_9/2_ → ^4^I_15/2_, respectively. As the concentration of Er^3+^ increased from 1 to 10 mol%, the green and blue emission intensities decreased, while the red emission intensity increased. This confirms the role of Er^3+^ in enhancing green and blue emission and suppressing red emission in NaYF_4_:Er^3+^/Mn^2+^ systems. These findings introduce the application of Mn^2+^ in Yb^3+^–Er^3+^ codoped NaYF_4_ UCNPs in color modulation, temperature sensing, and optical heating. The data presented in this study may provide useful information for further development of Ln-doped nanoparticles for these applications.

### Supplementary Information

Supplementary Information 1.

Supplementary Information 2.

## Data Availability

The datasets used and analyzed during the current study available from the corresponding author on reasonable request.
