# Supplementary material for: Understanding the effect of Mn2+ on Yb3+/Er3+ co-doped NaYF4 upconversion and obtaining the optimal combination of these tridoping
Source: Sci Rep. 2023 Oct 16;13:17556. doi: 10.1038/s41598-023-44947-1 (PMC10579380; doi:10.1038/s41598-023-44947-1)
Supplement: Supplementary file 2 — Supplementary Information 2. [file 41598_2023_44947_MOESM2_ESM.docx]

**2.2.2. Synthesis of NaYF_4_:5%Yb^3+^/x%Mn^2+^ (x%=20, 30, 40, 50, 60, 70) nanoparticles**

The synthesis of NaYF_4_:5%Yb^3+^/x%Mn^2+^ (x%=20, 30, 40, 50, 60, 70) nanoparticles was similar to that of NaYF_4_:5%Yb^3+^/30%Mn^2+^ nanoparticles. But the nanoparticles were exposed to the optimum synthesis temperature of 140 ° C for 8 hours.

**2.2.3. Synthesis of NaYF_4_:40%Mn^2+^/x%Yb^3+^ (x%=1, 5, 10, 20, 30, 40) nanoparticles**

The synthesis of NaYF_4_:40% Mn^2+^/x%Yb^3+^ (x%=1, 5, 10, 20, 30, 40) nanoparticles was similar to that of NaYF_4_:5%Yb^3+^/30%Mn^2+^ nanoparticles. But the nanoparticles were exposed to the optimum synthesis temperature of 140 ° C for 8 hours.

**2.2.4. Synthesis of NaYF_4_:2%Er^3+^/x%Mn^2+^ (x%=20, 30, 40, 50, 60, 70) nanoparticles**

The synthesis of NaYF_4_:2%Er^3+^/x%Mn^2+^ (x%=20, 30, 40, 50, 60, 70) nanoparticles was similar to that of NaYF_4_:5%Yb^3+^/30%Mn^2+^ nanoparticles. But the nanoparticles were exposed to the optimum synthesis temperature of 140 ° C for 8 hours.

**2.2.5. Synthesis of NaYF_4_:40% Mn^2+^/x%Er^3+^ (x%=1, 2, 5, 10) nanoparticles**

The synthesis of NaYF_4_:40% Mn^2+^/x%Er^3+^ (x%=1, 2, 5, 10) nanoparticles was similar to that of NaYF_4_:5%Yb^3+^/30%Mn^2+^ nanoparticles. But the nanoparticles were exposed to the optimum synthesis temperature of 140 ° C for 8 hours.

**2.2.6. Synthesis of NaYF_4_:40%Mn^2+^/1%Yb^3+^/x%Er^3+^ (x%=0, 2, 5, 10) nanoparticles**

The synthesis of NaYF_4_:40%Mn^2+^/1%Yb^3+^/x%Er^3+^ (x%=0, 2, 5, 10**)** nanoparticles was similar to that of NaYF_4_:5%Yb^3+^/30%Mn^2+^ nanoparticles. But the nanoparticles were exposed to the optimum synthesis temperature of 140 ° C for 8 hours.
